# *BACE1 *gene variants do not influence BACE1 activity, levels of APP or Aβ isoforms in CSF in Alzheimer's disease

**DOI:** 10.1186/1750-1326-5-37

**Published:** 2010-09-17

**Authors:** Annica Sjölander, Henrik Zetterberg, Ulf Andreasson, Lennart Minthon, Kaj Blennow

**Affiliations:** 1 Institute of Neuroscience and Physiology, Department of Neurochemistry and Psychiatry, Sahlgrenska University Hospital, University of Gothenburg, Sweden; 2 Clinical Memory Research Unit, Department of Clinical Sciences Malmö, Lund University, Sweden; 3 Neuropsychiatric Clinic, Malmö University Hospital, Sweden

## Abstract

The *BACE1 *gene encodes the beta-site APP-cleaving enzyme 1 and has been associated with Alzheimer's disease (AD). BACE1 is the most important β-secretase responsible for the generation of Alzheimer-associated amyloid β-proteins (Aβ) and may play a role in the amyloidogenic process in AD. We hypothesized that *BACE1 *gene variants might influence BACE1 activity or other markers for APP metabolism in the cerebrospinal fluid (CSF) and thereby contribute to the development of AD. We genotyped a Swedish sample of 269 AD patients for the rs638405 single nucleotide polymorphism (SNP) in the *BACE1 *gene and correlated genotype data to a broad range of amyloid-related biomarkers in CSF, including BACE1 activity, levels of Aβ_40_, Aβ_42, _α- and β-cleaved soluble APP (α-sAPP and β-sAPP), as well as markers for Alzheimer-type axonal degeneration, i.e., total-tau and phospho-tau_181_. Gene variants of *BACE1 *were neither associated with amyloid-related biomarkers, nor with markers for axonal degeneration in AD.

## Findings

Cleavage of the amyloid precursor protein (APP) by β- and γ-secretase gives rise to the amyloid β-protein (Aβ) found in senile plaques (SP) in Alzheimer's disease (AD). The *BACE1 *gene encodes the beta-site APP-cleaving enzyme 1 (OMIM 604252), which is involved in β-secretase activity [1-4]. The *BACE1 *gene is associated with AD [5,6] and the BACE1 activity is elevated both in brain tissue and in CSF in AD [7,8]. We hypothesized that *BACE1 *gene variants might influence the BACE1 activity or other amyloid-related biomarkers related to amyloid in the cerebrospinal fluid (CSF) and thereby contribute to developing AD. We tested a single nucleotide polymorphism (SNP) in the *BACE1 *gene to evaluate the genetic influence on BACE1 activity, levels of Aβ_40_, Aβ_42, _α- and β-cleaved soluble APP (α-sAPP and β-sAPP) in CSF from AD patients. Further we assessed the *BACE1 *genetic influence on markers for Alzheimer-type axonal degeneration (total-tau and phospho-tau_181_). The rs638405 SNP have a high allele frequency with a global frequency of the least common variant of 0.32. To our knowledge this is the first study investigates *BACE1 *genotype data in relation to BACE1 activity and other amyloid-related biomarkers in CSF from AD patients.

We studied a Swedish Caucasian sample of 269 AD patients (90 men and 179 women, mean age 74.7 ± 6.3 years) where CSF levels of total-tau, phospho-tau_181 _and Aβ_42 _were known. We measured the BACE1 activity, levels of α-sAPP, β-sAPP and Aβ_40 _in CSF samples from 84 of the patients. All participants were recruited at the Memory Clinic at the Malmö University Hospital. The patients gave informed consent to participate in the study, which was conducted according to the provisions of the Helsinki Declaration and approved by the local ethic committee. The diagnosis of "probable AD" was made according to the NINCDS-ADRDA criteria [9]. No AD patient had any of the known familial forms of autosomal dominant AD.

CSF samples were taken by lumbar puncture. BACE1 activity was determined with a sensitive and specific solution-based assay previously described [10]. Levels of total-tau, phospho-tau_181 _and Aβ_42 _were measured using established ELISA methods [11,12] while α-sAPP, β-sAPP and Aβ_40 _were quantified using MSD immunoassays (Cat#: K11120E and K111FTE), (Meso Scale Discovery, Gaithersburg, MD, USA).

Genomic DNA was extracted from whole blood using standard methods. *BACE1 *alleles were determined using the Dynamic Allele Specific Hybridization (DASH) technique as described earlier [13]. Optimal assay conditions: 1.5 mM MgCl_2_, 200 μM dNTPs, 0.05 U/μl Taq polymerase, 0.15 pmol/μl forward biotinylated primer (5'-Biotin-ATCCGGCGGGAGTGGTATTATG-3'), 0.75 pmol/μl reverse primer (5'-GTCCATTGATCTCCACCCGCAC-3') (Invitrogen, Life Technologies) and 5-20 ng DNA, 1xPCR buffer in a final volume of 25 μl. The cycling profile was: 5 min 95°C, 40 cycles: 30 sec 95°C, 45 sec 60°C, 1 min 72°C and a final step of 10 min 72°C. To identify *BACE1 *alleles the probes 5'-CACAATGATCACCTCATAA-3' and 5'-CACAATGATGACCTCATAA-3' were used.

*APOE *genotyping was performed using minisequencing as described before [14]. Gene designations follow the recommendations of HUGO Gene Nomenclature Committee [15].

The genotype and allele frequencies of the *BACE1 *rs638405 and *APOE ε4 *are shown in table 1. The analysis of variance (ANOVA) was used to analyze the effects of *BACE1 *genetic variants on MMSE, BACE1 activity and CSF protein levels. To test the effects of known risk factors, e.g., age, sex and *APOE ε4 *we identified significantly relevant covariates for each outcome variable (MMSE and levels of AD CSF biomarkers) using forward stepwise linear regression. Hardy-Weinberg equilibrium was assessed by χ^2 ^statistics. The criterion for significance was set at p < 0.05 for all statistical tests. Statistical analyses were performed with the SYSTAT11 (SYSTAT Software GmbH, Erkrath, Germany) software.

**Table 1:** *BACE1 *and *APOE *genotype and allele frequencies in AD patients

*BACE1*			
**Genotype frequencies**	**CC**	**CG**	**GG**
AD (269)	50 (0.19)	117 (0.43)	102 (0.38)

**Allele frequencies**	**C**	**G**	
AD (538)	217 (0.40)	321 (0.60)	

*APOE*			

**Genotype frequencies**	**No ε4**	**One ε4**	**Two ε4**
AD (269)	74 (0.28)	135 (0.50)	60 (0.22)

**Allele frequencies**	**ε2/ε3**	**ε4**	
AD (538)	283 (0.53)	255 (0.47)	

Abbreviations: Alzheimer's disease (AD). Number of total genotypes and alleles are given (N). Percentage of total is shown for the genotypes and alleles respectively.

We studied *BACE1 *gene variants in relation to BACE1 activity and levels of amyloid-related biomarkers in CSF from AD patients. In the linear regression analysis we found *APOE ε4 *to significantly interact with phospho-tau18, Aβ_42 _and MMSE. Subsequently, *APOE ε4 *was included as covariate in the statistic model. We found no effect of *BACE1 *gene variants on BACE1 activity or levels of Aβ_40_, Aβ_42, _α-sAPP and β-sAPP (Table 2). We compared CSF levels of markers for axonal degeneration (total-tau and phospho-tau_181_) between *BACE1 *gene variants and found no significant differences in protein levels (Table 2). The *BACE*1 SNP showed no association with MMSE (data not shown).

**Table 2:** BACE1 activity and biochemical markers in CSF and *BACE1 *genotypes in AD patients

*BACE1 *genetic variants				
CSF protein	CC	CG	GG	p-value
Total-tau (pg/ml)	628.8 ± 38.7	612.7 ± 29.3	610.0 ± 33.4	0.699
Phospho-tau_181 _(pg/ml)	79.7 ± 3.9	73.8 ± 2.8	77.7 ± 3.4	0.506
Aβ_42 _(pg/ml)	408.7 ± 11.8	419.4 ± 9.2	408.3 ± 9.9	0.575
Aβ_40 _(pg/ml)	4869.9 ± 196.4	4894.8 ± 238.6	5030.7 ± 219.6	0.854
BACE1 (pM)	29.2 ± 2.5	31.8 ± 2.0	33.6 ± 3.3	0.765
α-sAPP (ng/ml)	645.8 ± 77.0	660.5 ± 40.6	620.5 ± 41.8	0.962
β-sAPP (ng/ml)	393.3 ± 37.8	408.0 ± 24.9	399.6 ± 22.6	0.965

Abbreviations: Alzheimer's disease (AD). Mean values (± SEM) are shown. Adjusted p-values are shown for respective comparison.

The *APOE *allele distribution followed the expected frequencies seen in AD populations (Table 1). Genotype frequencies conformed to Hardy-Weinberg equilibrium in AD patients.

We hypothesized that *BACE1 *gene variants might influence BACE1 activity or levels of amyloid-related biomarkers in the cerebrospinal fluid (CSF) and thereby contribute to the development of AD. A genome-wide screen of AD families has shown linkage to marker close to *BACE1 *[16] and the rs638405 SNP has been tested in a number of studies [5,17]. Other studies have found association between the *BACE1 *gene and AD [5,18]. Even though this SNP does not change the protein sequence it still can have functional effects based on earlier findings where *BACE1 *influenced levels of Aβ in CSF [6]. We tested if the rs2069456 SNP was associated with BACE1 activity or with levels of Aβ_40_, Aβ_42, _α-sAPP and β-sAPP in CSF from AD patients. Further we assessed the influence on total-tau and phospho-tau_181_. Gene variants of *BACE1 *were neither associated with amyloid-related biomarkers nor with markers for axonal degeneration. Our results do not support that variants of the *BACE1 *gene affects BACE1 activity or CSF levels of APP or Aβ isoforms in AD.

## Competing interests

The authors declare that they have no competing interests.

## Authors' contributions

AS participated in the design of the study, performed the genetic analyses, analyzed the data statistically and drafted the manuscript. HZ participated in the design of the study, helped in analyzing the data and helped in drafting the manuscript. UA performed the activity analyses and the immunoassays and revised the manuscript critically. LM collected the clinical material and revised the manuscript critically. KB participated in the design of the study, helped in analyzing the data and revised the manuscript critically. All authors read and approved the final manuscript.
